# Dynamic regulation of aquaporin-4 water channels in neurological disorders

**DOI:** 10.3325/cmj.2015.56.401

**Published:** 2015-10

**Authors:** Ying Hsu, Minh Tran, Andreas A. Linninger

**Affiliations:** Laboratory for Product and Process Design, Department of Bioengineering, University of Illinois at Chicago, Chicago, IL, USA

## Abstract

Aquaporin-4 water channels play a central role in brain water regulation in neurological disorders. Aquaporin-4 is abundantly expressed at the astroglial endfeet facing the cerebral vasculature and the pial membrane, and both its expression level and subcellular localization significantly influence brain water transport. However, measurements of aquaporin-4 levels in animal models of brain injury often report opposite trends of change at the injury core and the penumbra. Furthermore, aquaporin-4 channels play a beneficial role in brain water clearance in vasogenic edema, but a detrimental role in cytotoxic edema and exacerbate cell swelling. In light of current evidence, we still do not have a complete understanding of the role of aquaporin-4 in brain water transport. In this review, we propose that the regulatory mechanisms of aquaporin-4 at the transcriptional, translational, and post-translational levels jointly regulate water permeability in the short and long time scale after injury. Furthermore, in order to understand why aquaporin-4 channels play opposing roles in cytotoxic and vasogenic edema, we discuss experimental evidence on the dynamically changing osmotic gradients between blood, extracellular space, and the cytosol during the formation of cytotoxic and vasogenic edema. We conclude with an emerging picture of the distinct osmotic environments in cytotoxic and vasogenic edema, and propose that the directions of aquaporin-4-mediated water clearance in these two types of edema are distinct. The difference in water clearance pathways may provide an explanation for the conflicting observations of the roles of aquaporin-4 in edema resolution.

## Introduction: aquaporin-4 water channels in neurological disorders

Aquaporin-4 (AQP4) channels are the most ubiquitous water channels in the central nervous system (CNS). They are bidirectional water conduits highly concentrated in astrocytic endfeet (1) and glial limitans (2). AQP4 channels play important roles in neurological disorders. The importance of AQP4-mediated water flux in potassium homeostasis is established in epilepsy (3,4). In human epilepsy, a defect in erythrocyte membrane water permeability is found, suggesting a global mechanism of defective membrane water permeability (5). Importantly, even though AQP4 channels are concentrated in glial cells, their role in brain homeostasis is increasingly linked to neuronal survival. Failure of brain homeostasis maintained by glial cells has been postulated to underlie neuronal cell death in amyotrophic lateral sclerosis (ALS), and an up-regulation of AQP4 has been found in a rat model of ALS (6). AQP4 levels are up-regulated in the frontal cortex of patients with prion disease, likely in response to the disturbed water homeostasis leading to the swelling of neuronal and astrocytic processes (7). In addition to their role in brain water transport and ionic homeostasis, AQP4 channels have been shown to influence the clearance of proteins from the brain parenchyma, including β-amyloid (8). These emerging studies support that AQP4-mediated water transport strongly influences the clearance of metabolites and ions in the brain. AQP4 channels are attractive therapeutic targets not only for their role in brain water homeostasis, but also for their impact on the clearance of molecules from the parenchyma.

Manipulating AQP4 expression levels in astrocytes can alter cell membrane water permeability (9-11). Beyond the cellular level, Badaut et al (12) showed that gene silencing of *Aqp4* in rat decreased the apparent water diffusion coefficient by 50% measured with diffusion-weighted imaging (DWI). The expression of AQP4 not only alters the water permeability of cell membranes in culture, but also regulates the water permeability of the brain. However, the route of AQP4-mediated water transport in the brain is not clearly understood.

AQP4 expression levels and sub-cellular localization both exhibit dynamic spatiotemporal patterns after neurological injury. It has been shown that cerebral edema causes a dynamic change in AQP4 levels, and these levels correlate with the apparent water diffusivity in the brain (13). On the other hand, evidence suggests that perivascular AQP4 expression is a rate-limiting factor in edema formation (14). Accordingly, different rates and severity of edema formation have been found between control animals and animals with altered AQP4 expression using genetic knockout (15) or glial-specific overexpression (16). In addition, an astrocyte-specific conditional knockout model of *Aqp4* (*cAqp4* KO) in mice provides evidence that brain water entry during cytotoxic edema is mediated by AQP4 channels in astroglial cells. Forty minutes after intraperitoneal water injection, *cAqp4*-KO mice have 31% less brain water content (17). These studies support that AQP4 located in astroglial cells plays a critical role in brain water regulation in edema.

However, it has been found that AQP4 channels in edema can play either a beneficial or detrimental role. A detrimental effect of AQP4 in cytotoxic edema has been observed using *Aqp4* knockout mice (15). On the other hand, compared to *Aqp4*-null mice, wild type mice have better survival in vasogenic edema (18). The reason behind the opposing role of AQP4 is currently an area of intense debate. Understanding the complex mechanism of brain water transport at the cellular level is critical in order to effectively develop and successfully administer molecular therapeutics (19). In this review, we propose the hypothesis based on current evidence that whether AQP4 is beneficial or detrimental in a certain type of edema depends on the direction of osmotic gradients between the cell, the extracellular space, and the blood. We will support this hypothesis with published findings on ionic concentrations in the brain after injury and propose directions of AQP4-mediated water transport. Recently, a pharmacological blocker of AQP4, which is also an inhibitor of Na^+^-K^+^-2Cl^-^ cotransporter, has been shown to reduce edema post-ischemia at the 24-hour time point; however, the beneficial effect is reversed at 48 hours (20). The reversal of the beneficial effect could possibly reflect the difference in water transport pathways at the cellular level during cytotoxic and vasogenic edema. These studies highlight the need for a clearer understanding of the microscopic route of water clearance in the brain, and the direction of water transport mediated by AQP4 between the cytosol, extracellular space, cerebrospinal fluid (CSF), and blood. The purpose of this review is to address this open area of debate as it relates to the role of AQP4 channels in brain water clearance in neurological disorders.

This review begins with the examination of changes in AQP4 expression levels in neurological conditions. Then, we will analyze whether the change in AQP4 expression and sub-cellular localization post-injury is favorable or deleterious. Based on the osmotic measurements in the brain during edematous conditions, we propose the direction of AQP4-mediated water transport in both cytotoxic and vasogenic edema, and highlight the critical difference. The review will end with recommendations for developing therapies to promote brain water clearance.

## Aquaporin-4 expression dynamics in brain injury

AQP4 expression in the brain after neurological injury shows a temporally dynamic as well as spatially heterogeneous pattern. This complex temporal evolution is likely caused by multiple regulation mechanisms at both the transcriptional and post-transcriptional levels in response to signaling events activated by secondary injuries. Most data contain measurements of AQP4 protein or mRNA levels in the entire brain or in selected regions of bulk brain tissue. However, AQP4 channels on the membrane are assembled into square arrays (21), and the formation of square arrays impacts the membrane water permeability. The size of the square arrays is controlled by the ratio of the two principle isoforms of AQP4: the M23 isoform and the M1 isoform (21). Therefore, astrocytes can control membrane water permeability through regulating the expression ratio of AQP4 isoforms. Recently, the sub-cellular localization and oligomeric disruptions of AQP4 after neurological injury have gathered attention. Dynamic changes of AQP4 levels in specific cellular pools (such as localization between endfeet vs non-endfeet membranes, membrane-bound vs within intracellular vesicles, and in oligomeric form vs disrupted arrays) can offer significant insight into the route of brain water clearance.

**Figure 1:**
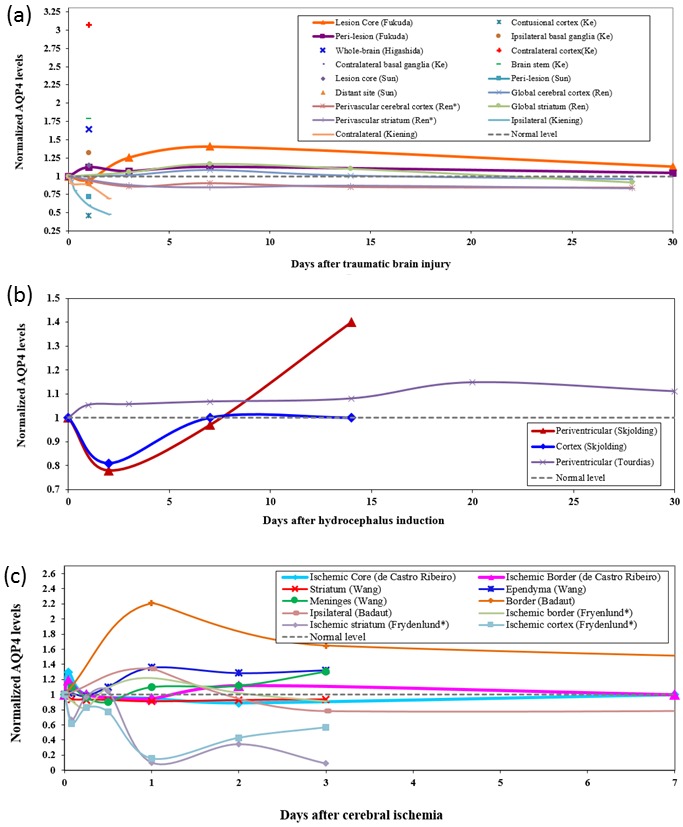
Dynamics of aquaporin-4 (AQP4) expression levels after (**A**) experimental traumatic brain injury (TBI), (**B**) hydrocephalus, and (**C**) cerebral ischemia. (**A**) Fukuda et al (28) quantified AQP4 levels by immunoreactivity in a rat model of juvenile TBI. Higashida et al (22) measured AQP4 levels in whole-brain lysates with western blotting in a closed-head injury model of rat. Ke et al (26) and Sun et al (24) measured *Aqp4* mRNA levels with reverse transcription-polymerase chain reaction in rats. Ren et al (23) measured both global AQP4 levels as well as changes in perivascular AQP4 polarization and found a loss of AQP4 polarization despite a slight global increase. Kiening et al (29) measured AQP4 levels with immunoblotting in ipsilateral and contralateral hemispheres in rats. Subcellular region-specific measurements are denoted with asterisk. Results are shown as fold change compared to the control group. (**B**) Temporal expression levels of AQP4 after the induction of hydrocephalus in rats. Skjolding et al (37) quantified AQP4 levels over 14 days in the cortex and periventricular zones by western blotting. Using a different model of hydrocephalus, Tourdias et al (38) also observed an elevation of AQP4 in the periventricular region. This increase could have implications in brain water clearance. (**C**) Temporal dynamics of AQP4 expression levels in the brain after experimental models of ischemia. AQP4 levels after transient focal ischemia were measured by western blotting in mice by de Castro Ribeiro et al (43). The region-specific expression of AQP4 often characterized by an initial increase within a few hours of primary injury was also measured by Wang et al (13) using a transient global ischemia model in piglets. Except in the ischemic core and striatum, AQP4 levels displayed a delayed but significant upregulation 12 to 24 hours after ischemia. Badaut et al (45) quantified AQP4 immunoreactivity in a neonatal rat model of middle cerebral artery occlusion. Frydenlund et al (47) quantified perivascular AQP4 with immunogold labeling in mice subject to 90 minutes of middle cerebral artery occlusion.

First, experimentally measured AQP4 trajectories in animal models of traumatic brain injury (TBI), hydrocephalus, and ischemia are summarized in Figure 1. These patterns illustrate the high degree of heterogeneity in post-injury expression patterns in the brain. Is the induced change in AQP4 levels a mere consequence of pathological signaling, or does it constitute a beneficial and protective mechanism? What are the regulatory mechanisms at work to control the acute vs long term change in brain water permeability? Summaries of AQP4 measurements in the brain in traumatic brain injury, hydrocephalus, and ischemia are presented, followed by an analysis of the change in the osmotic environment in the brain, the activation of cellular signaling, and the proposed hypothesis regarding the direction of water transport post-injury.

### Traumatic brain injury

AQP4 exhibits a complex spatiotemporal dynamics after TBI, and conflicting evidence are reported. In a closed-head model of TBI, AQP4 is found to be up-regulated by more than 50% in the lysates of the whole brain 24 hours after injury, accompanied by dextran extravasation, which is indicative of vasogenic edema (22). In another closed-head model of the TBI, global AQP4 immunoreactivity in both the cortex and striatum is found to be increased up to 14 days post-injury (23). However, the location-specific quantification of perivascular AQP4 in these areas shows a reduction in AQP4 immunoreacivity up to 28 days post injury, highlighting the difference in AQP4 global expression level and its subcellular localization. In this study, the “polarization index” of AQP4 in glial cells decreases post-injury, which seems to indicate that the reduction in perivascular AQP4 post-injury is due to its redistribution to other parts of the cell, accompanied by a global increase in AQP4 level. The functional consequence of AQP4 membrane relocalization is unclear. However, the change in its sub-cellular localization can be a mechanism to rapidly control water flux, compared to the slower, longer-lasting transcriptional regulation.

In a rat model of TBI with focal cortical contusion, mRNA of *Aqp4* is up-regulated in the lesion core, but down-regulated in the periphery of the lesion core (24). Interestingly, the levels of microRNA-320, which was shown to regulate *Aqp4* transcripts, show opposite trends to *Aqp4* mRNA levels, indicating the possibility of post-transcriptional regulation of AQP4 after TBI. More specifically, microRNA-320 was dramatically reduced in the contusion cortex, while markedly up-regulated around the injury core (25). On the contrary, Ke et al (26) found that the expression of AQP4 protein diminished almost completely in the contusional cortex at 1 day post injury, while the mRNA was decreased to 50% compared to sham-operated animals. The near complete disappearance of AQP4 proteins but not mRNAs confirms possible translational repression mechanisms by elements such as microRNAs or the regulation of AQP4 protein stability. The translational repression may be a protective response to regulate the existing pool of *Aqp4* transcripts in the cell at the time of injury. At the contusional core, vasogenic edema characterized by compromised BBB integrity was found. After controlled cortical impact injury, Guo et al (27) found no significant changes in AQP4 expression at 24 hours compared to sham animals. After 72 hours, AQP4 expression increased in the pericontusional area to 175% of control and to 200% of control near the lateral ventricles. In a rat model of juvenile TBI, AQP4 levels diminished by about 5% at the lesion core at 1 day post injury, but increased by about 40% within 7 days. In the peri-lesion areas, a slight elevation of AQP4 immunoreactivity between 0%-20% was observed within a 30-day period (28). Kiening et al (29) measured AQP4 in the ipsilateral and contralateral hemispheres using immunoblotting after controlled cortical impact injury. AQP4 decreased in both hemispheres throughout 48 hours, without distinguishing the contusional core and pericontusional areas. In this particular TBI model, cytotoxic edema was found to prevail during the early phases post-injury (30). These studies are summarized in Figure 1, in which measurements at discrete time points over time from the same study were connected to better depict the temporal trajectory.

The wide disparity between these reported AQP4 expression levels shows that expression patterns of AQP4 after experimental TBI are not only specific to the injury model used, but also highly dependent on the regions of analysis. It has been noted that differences in secondary injury mechanisms exist between TBI models with open or closed cranium, since an elevated intracranial pressure will not develop in a TBI model using an open cranium, leading to different injury dynamics (23). Importantly, findings also varied between methods of measurement depending on whether the method distinguished different sub-cellular pools of AQP4 (immunofluorescence vs Western blotting). In addition, the mRNA levels do not reflect the changes in protein levels, pointing toward the importance of translational and post-translational regulatory mechanisms at work post-injury. Therefore, both mRNA and protein should be assayed to unveil the regulatory mechanisms that lead to the heterogeneity and temporal dynamics of AQP4. Despite these variations, a common observation is an elevation of AQP4 levels in the peri-lesion areas after TBI, which was found to persist even after one month. After TBI, diffusion-weighted imaging shows that both cytotoxic and vasogenic edema are present (31). Zhao et al (32) shows that after TBI, sulforaphane-administered animals have reduced edema, and this beneficial effect could be due to the elevation of *Aqp4* mRNA and protein levels by sulforaphane administration. In animal models of TBI, the sustained elevation of AQP4 levels in the peri-lesion areas could be due to transcriptional up-regulation caused by neuroinflammation. Inflammatory cytokines such as interleukin-1 (IL-1) are found to be inducers of AQP4. The release of interleukin-1 (IL-1) (33), tumor necrosis factor-α (TNF-α) (34), IL-6 (34), and excitotoxic glutamate (34) has been found in rodent models of TBI. Elevation of IL-1 is found in the cerebrospinal fluid (CSF) of patients with severe head injury (35). The hypothesis that the region-specific AQP4 expression in the brain after injury serves as a mechanism for fluid drainage remains a possibility.

### Hydrocephalus

In hydrocephalus, excess CSF accumulates in brain ventricles, causing periventricular edema, deep white matter ischemia, and a decrease in gray matter extracellular space (ECS) volume fraction due to brain tissue compression (36).

The molecular mechanisms driving the accumulation of cerebrospinal fluid in hydrocephalus are poorly understood, but it is plausible to investigate the role of AQP4 channels in facilitating fluid exchange between the capillary bed and the extracellular space. During the onset of kaolin-induced hydrocephalus, AQP4 levels in the periventricular region were decreased at 48 hours, followed by a gradual recovery. Normal level was reached about 7-8 days post induction, and afterwards a continuous increase in AQP4 was found in the periventricular region, whereas the normal level was maintained in the cortex (37). In a rat hydrocephalus model using L-α-lysophosphatidylcholine stearoyl injection, a continuous elevation of AQP4 in the periventricular region was seen throughout 30 days. These dynamic trajectories are shown in Figure 1. This observed increase of AQP4 probably underlies the elevation in brain water permeability as measured by diffusion tensor imaging in hydrocephalic rats (38). During progressive hydrocephalus, the extracellular space is observed to enlarge in the periventricular region. In cortical layer I, the extracellular space is reduced from 16.5% of brain volume in normal rats to 9.6% in hydrocephalic rats (36).

In both studies, a sustained elevation in AQP4 expression in the periventricular region was observed. Since excess CSF accumulates within the brain ventricles, the elevation of AQP4 in the periventricular region could be a protective mechanism for facilitating brain water clearance. Indeed, a subset of *Aqp4*-null mice developed obstructive hydrocephalus with elevated intracranial pressure (39). In addition, in a kaolin-induced hydrocephalus model, hydrocephalic *Aqp4*-null mice showed increased mortality rate, elevated intracranial pressures, and higher brain water content after kaolin induction compared to wild-type mice (40). The study further conjectured that the up-regulation of AQP4 would facilitate water clearance in hydrocephalus (40). Together, these studies suggest that the observed AQP4 up-regulation in the periventricular area is protective. This up-regulation is likely due to the transcriptional up-regulation of the gene caused by neuroinflammation. In hydrocephalus, high levels of inflammatory cytokines such as TNF-α (41) and IL-1β (42) are found in the CSF. The periventricular up-regulation of AQP4 may point to the existence of a water clearance pathway mediated by astrocytic endfeet near the ventricles.

### Cerebral ischemia

Cerebral ischemia is a condition characterized by reduced blood flow and oxygen supply due to an occlusion event. Transient or permanent ischemia induces changes in the brain microenvironment such as ATP depletion, oxidative stress, accumulation of ions, and leads to a shift in osmotic gradients from the normal homeostasis. These changes activate signaling cascades that alter gene expression levels and promote inflammation.

AQP4 expression after ischemic injury also shows spatiotemporal heterogeneity. In a transient focal ischemia model with 30 minutes of occlusion followed by reperfusion, AQP4 expression was found to increase at 1 hour, returned to normal levels at 6 hours and 24 hours, and decreased at 48 hours at the ischemic core. In the border zone, similar patterns are observed at 1, 6, and 24 hours; however AQP4 expression increased at 48 hours (43). In another study, immunohistochemical staining showed that AQP4 levels were nearly abolished at the ischemic core after 2 hours of middle cerebral artery occlusion and 4 hours of reperfusion, even though the total amount of AQP4 determined by Western blot was unchanged (44). After hypoxic/ischemic injury followed by reperfusion in piglets, Wang et al (13) found elevated expression of AQP4 in regions near fluid spaces including ependyma and meninges, and decreased expression in the striatum. Badaut et al (45) quantified AQP4 immunoreactivity in a neonatal rat model of middle cerebral artery occlusion, as well as used MRI to assess changes in ADC values and T2 values to track the development of cytotoxic and vasogenic edema. A significant elevation of AQP4 in the border zone was observed, where AQP4 was concentrated in the perivascular spaces. It was hypothesized that the elevation of AQP4 in this region assisted in the clearance of edema fluid (45).

Following up on the results by Ribeiro et al (43), the study by Hirt et al (46) determined that AQP4 rise one hour after ischemia was mainly due to the increase in the M1 isoform, while the M23 isoform experienced a small increase that did not reach the significance level. The increase in M1 isoform is also found at the mRNA level, whereas the mRNA for the M23 isoform cannot be detected (46). Ischemia produces a rapid response in promoting the expression of the M1 isoform, while reducing the expression of the M23 isoform, likely to decrease the formation of square arrays on the cellular membrane of astrocytes. Interestingly, this observation is in line with findings by a later study that shows the disruption of AQP4 square arrays in the perivascular endfeet membrane after ischemia (47). These studies show that the isoform-specific control of AQP4 expression is important and may have consequences in regulating membrane water permeability.

More specifically, AQP4 expression in different sub-cellular pools was examined after middle cerebral artery occlusion followed by reperfusion. The disruption of AQP4 square array formation on the membrane 30 minutes after ischemia was observed (48,49). Frydenlund et al (47) showed that there was a loss of AQP4 in the perivascular pool at the injury core after transient ischemia in a mouse model. At 24 hours and 48 hours, there was a 78% and 58% loss of AQP4 staining around the perivascular endfeet of astrocytes at the neocortical lesion area. This loss was accompanied by disruption of AQP4 square arrays revealed by freeze fracture microscopy (47). Given that the maximal brain water content was found at 48 hours, and the deletion of perivascular AQP4 was found to alleviate edema formation after ischemia (14), the loss of AQP4 at the endfeet surrounding capillaries may be a protective response of the brain to reduce edema formation. Together with the findings by Hirt et al (46), this suggests that the protective reduction of endfeet water permeability near the vasculature could be jointly regulated by the transcriptional control of the M1 and M23 isoforms along with the disruption of existing square arrays. Interestingly, while a loss of AQP4 near the vasculature at the injury core is observed, a slight increase is seen in the border zone. In accordance with the loss of perivascular AQP4, disruption of square arrays in the injury core is observed while abundant square arrays are seen at the endfeet in the border zone (47). To examine whether the perivascular AQP4 pool has an effect on brain water egress, brain water content was measured in α-syn^−/−^ mice (syntrophin deletion predominantly affects the anchoring of AQP4 on the perivascular endfeet membrane) and wild type (WT) mice after transient cerebral ischemia. The brain water content 48 hours after ischemia was higher in WT mice than in α-syn^−/−^ mice. Since α-syn^−/−^ mice lack perivascular AQP4, this confirms that the removal of perivascular AQP4 has a protective effect on edema. The different severity of edema in wild-type and syn^−/−^ mice suggests that the perivascular pool of AQP4 is a rate-limiting factor on edema formation (14). In addition, the perivascular AQP4 pool is responsible for the difference in the severity of cytotoxic edema in distinct brain regions following water intoxication (50). Similarly, treatment of mice after middle cerebral artery occlusion with bumetanide, which inhibits Na^+^-K^+^-2Cl^-^ cotransporter and recently has been found to be a blocker of AQP4, attenuates edema in wild type mice but has no effect of α-syn^−/−^ mice at 24 hours, further validating the role of perivascular AQP4 in edema formation (20). These studies confirm that the perivascular pool of AQP4 is rate-limiting for brain water entry in cytotoxic edema and the rapid disruption of square arrays is a protective mechanism.

Interestingly, the perivascular pool of AQP4 at the astroglial endfeet also mediates water clearance in cerebral edema in addition to edema formation. Cerebral edema can be alleviated by elevating blood osmolarity through the intravenous infusion of a hypertonic solution. Treatment with an intravenous infusion of hypertonic saline attenuated brain edema in WT mice, but had no effect on syn^−/−^ mice, demonstrating that hyperosmolar blood promotes brain water egress mediated through the perivascular AQP4 pool in astrocytes (14). These studies illustrate that AQP4 mediates brain water entry and egress at the blood-brain interface, and the regulation of AQP4 anchoring at this site can accelerate or slow water entry or clearance. These findings highlight the importance of studying AQP4 sub-cellular localization in order to elucidate the cellular pathway of water transport between blood, brain tissue, interstitial fluid, and CSF.

The dynamic measurements of AQP4 levels in ischemia are represented in Figure 1. Data accounting for the sub-cellular localization of AQP4 are denoted with an asterisk. At the core, AQP4 expression is either unchanged or decreased after a few hours. Despite the variability between studies, the data agree on the observation that elevated AQP4 expression is found at the border zone at the 24-hour time point. This increase coincides with the approximate timing of BBB opening, which is generally believed to occur between 6-24 hours after injury.

## Regulation of brain water transport in cerebral ischemia

**Figure 2:**
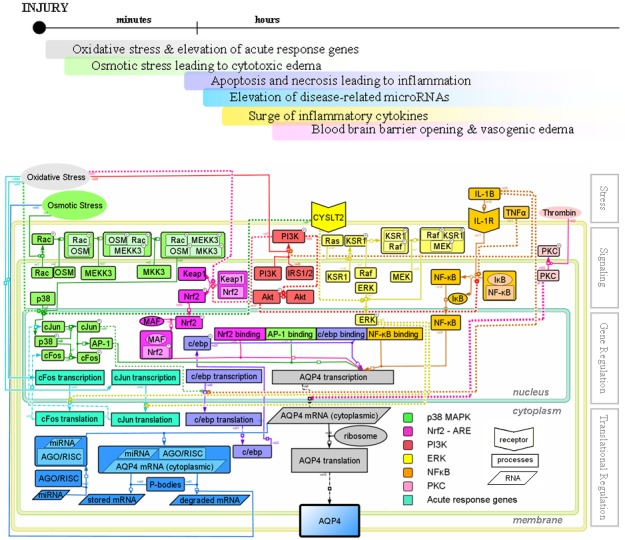
Signaling and regulation of aquaporin-4 expression after ischemic injury. Top frame shows the temporal phases of secondary injury mechanisms following ischemia. The astrocyte cell is divided into three compartments: nucleus, cytoplasm, and membrane. The membrane compartment is designed to illustrate the translocation of signaling molecules towards the membrane. The upper portion of the cell shows signaling events during injury leading to the transcriptional regulation of the aquaporin-4 gene. The center portion shows known transcription factor binding sites on the promoter of aquaporin-4 gene. The lower portion shows translational regulation events by microRNAs. The extracellular insults above the cell are arranged according to the approximate timeline of secondary injuries after cerebral ischemia. “P” within a circle marking the upper-right corner of certain molecules represents the phosphorylated state, and dashed lines indicate that the detailed signaling mechanism is still unknown. Arrows indicate activation, while flat caps indicate inhibition. The figure is generated in CellDesigner (153).

We will discuss the role of AQP4 in injury mechanisms including oxidative stress, ionic dysregulation, edema, and inflammation. In the literature, there are an abundance of reports investigating the regulation of the *Aqp4* gene by signaling. However, these independent investigations need to be summarized to obtain a comprehensive understanding of AQP4 gene regulation. The signaling pathways activated during these injury mechanisms and their reported effect on the *Aqp4* gene are represented in a signaling map (Figure 2). This map enhances the analysis of how these signaling pathways jointly regulate *Aqp4* in the injury state. We will discuss the complex regulatory mechanisms at work to control *AQP4 *expression. Furthermore, we propose the directions of AQP4-mediated water flux in cytotoxic and vasogenic edema based on their osmolar environment, and elucidate their mechanisms of brain water clearance.

### Causes of oxidative stress and its influence on AQP4 expression

An interruption in blood supply leads to hypoxia and decreased ATP synthesis by the mitochondria. In transient ischemia, initial oxygen depletion due to the cessation of blood supply is followed by a sudden rise in oxygen tension during blood reperfusion. Reperfusion in effect accelerates harmful oxidizing reactions (51). During normal cellular processes, a low level of reactive oxygen species (ROS) is generated in the mitochondria. However, during oxidative stress, abnormally high levels of ROS can induce apoptosis (52) and necrosis (53). The excess generation of ROS occurs not only after ischemic-reperfusion injury, but also after TBI (54-57), traumatic spinal cord injury (58), and hemorrhage (59). Oxidative stress is a common mechanism of secondary injury.

The induction of oxidative stress is followed by a rapid elevation of immediate early genes such as *c-Jun* and *c-Fos* (60,61), which is depicted in Figure 2. Together, c-FOS and c-JUN form the activating protein-1 (AP-1), a target of the p38 mitogen activated protein kinase (MAPK) pathway. Activation of the p38 MAPK pathway is known to induce transcriptional up-regulation of AQP4 (62). Oxidative stress activates p38 within 5 minutes in astrocytes (63). Together, evidence suggests that oxidative stress can rapidly induce the expression of AQP4. It could also be the cause of the up-regulation of *Aqp4* mRNA in animal models.

Oxidative stress also activates a key transcription factor for inflammatory response, nuclear factor κB (NFκB) (64-66), which is a putative transcription factor for the *Aqp4* gene (67). In addition, oxidative stress activates genes containing the antioxidant response element (ARE) via NFE2-related factor-2 (NRF2). The nuclear translocation of NRF2 requires the reorganization of actin controlled by the PI3K signaling pathway (68,69). The promoter region of *Aqp4* gene contains putative AREs, and sulforaphane, a known activator of NRF2, has been found to up-regulate AQP4 in vivo (32,70). The effectiveness of sulforaphane in vivo illustrates the importance of understanding the signaling mechanisms controlling *Aqp4* gene expression.

In addition, oxidative stress has been shown to induce *AQP4* expression through the production of pro-inflammatory leukotrienes. Oxidative stress stimulates the release of arachidonic acid (AA) from the cellular membrane (71), and leukotrienes are produced as a metabolite of AA (72-74). They are released into the brain after TBI (75,76), fluid-percussion injury (77), ischemia (73,78), and hemorrhage (78-80). Leukotrienes play an important role during the inflammatory response in inducing leukocyte-endothelial adhesion and increasing BBB permeability (81). Activation of cysteine leukotriene CysLT_2_ receptor induces AQP4 up-regulation through the p38 and ERK signaling pathways in astrocytes (82), as shown in Figure 2. The up-regulation of AQP4 by leukotrienes suggests that inflammatory mediators like leukotrienes could potentially cause a dynamic change in water permeability at the BBB in addition to their role in increasing macromolecule permeabilization.

In summary, evidence shows the direct regulation of *Aqp4* gene expression through various transcription factors activated by oxidative stress. At this early phase, the increase in AQP4 is likely to be harmful due to the formation of cytotoxic edema. Indeed, Piroxicam-induced neuroprotection in ischemic-reperfusion injury is mediated by the down-regulation of AQP4 (83). There is a well-known steroid mediated neuroprotection in stroke (84). Administration of progesterone in TBI patients improved survival, and lowered intracranial pressure (85), indicating that progesterone may aid in brain water clearance. Interestingly, application of progesterone on glial cell cultures inhibited oxidative stress-induced AQP4 expression (86). The modulation of AQP4 expression has functional consequences in conferring neuroprotection.

### Formation and clearance of cytotoxic edema

**Figure 3:**
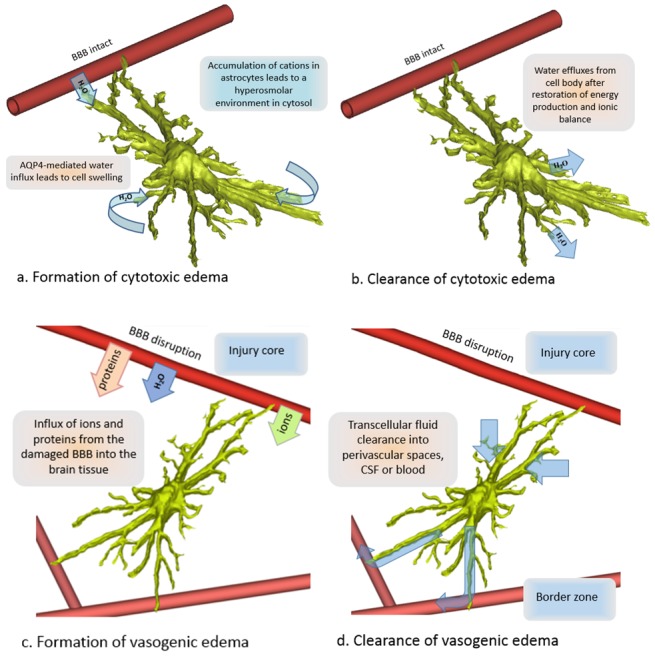
The proposed direction of water transport during the formation and resolution of cytotoxic edema (**A,B**) and vasogenic edema (**C,D**). Ischemia-induced hypoxia causes a failure in energy-dependent ion pumps such as Na^+^-K^+^-ATPase, leading to accumulation of cations in the cell. Aquaporin 4 (AQP4)-mediated water influx aggravates cytotoxic cell swelling (**A**). When energy-dependent ion pumps are restored, the cytosol and the extracellular space return to osmotic homeostasis by active ion transport and the passive efflux of water from the cell (**B**). Between 6-24 hours after ischemic injury, the disruption of the blood brain barrier, as well as sodium influx into the brain at the injury core cause vasogenic edema (**C**). Water clearance accompanies the clearance of osmolytes, partially through the transcellular route (**D**). The cell was reconstructed from a stack of confocal images of a single astrocyte. We propose that the opposing roles of AQP4 are explained by the difference in osmotic gradients between blood, extracellular space, and intracellular space during cytotoxic and vasogenic edema as well as the distinct routes of osmolyte clearance.

Reduced blood flow causes hypoxia and ATP depletion. ATP depletion leads to the failure of energy dependent ion pumps, which severely disrupts the osmotic balance in the brain (87). The concentration of potassium ions in the extracellular space ([K^+^]_ex_) rapidly increases after ischemia (88,89) due to the failure of pumps such as the Na^+^-K^+^ ATPase, which exchanges three intracellular sodium ions with two extracellular potassium ions in normal physiological conditions. Correspondingly, the concentration of intracellular K^+^ decreases while Na^+^ accumulates. The magnitude of increase in [Na^+^] is 2.4 fold of the magnitude of decrease in [K^+^], as measured 4 hours after permanent occlusion in rats (90). The accumulation of excess ions within the cytosol causes a hyperosmolarity inside the cell, which induces cell swelling and cytotoxic edema (87). Astrocyte swelling caused by a hyperosmolar cytosol is demonstrated in Figure 3A. A significant intracellular increase in Na^+^ accompanied by shrinking of extracellular space from 18.9% to 8.5% of cortical volume was detected within 60 minutes of global ischemia (87). Indeed, increased brain water content at the ischemic core indicates the formation of edema as early as 1 hour after the occlusion event (91). Interestingly, this ionic imbalance is recovered rapidly once the blood flow is restored, indicating that cytotoxic edema is tightly coupled to energy deficiency (89). The clearance of cytotoxic edema by re-establishing ionic and water balance is illustrated in Figure 3B. In addition, ionic imbalance induces disregulation of neurotransmitters. Elevation of [K^+^]_ex_ evokes glutamate release into the extracellular space (88). Intriguing evidence was found regarding the regulation of AQP4 permeability through direct gating mechanism by the activation of glutamate receptors (92), but this evidence was recently contradicted (93).

AQP4 expression is a rate-limiting factor in the formation of cytotoxic edema. Cytotoxic edema is marked by the preferential swelling of astroglial cells (94), which could be attributed to their expression of AQP4. The detrimental role of AQP4 during the development of cytotoxic edema is demonstrated by *Aqp4*-knockout mice (15), consistent with the view that AQP4 on astroglial cells promotes cell-swelling during this stage (16). Interestingly, as we have discussed previously, the brain seems to have in place a physiological mechanism to counteract the harmful role of AQP4 by the action of microRNAs, despite the induction of its transcription at these early times. Furthermore, the disruption of square arrays provides yet another mechanism of protection. We suggest that the induction of *Aqp4* transcripts by ROS is a consequence of signaling, whereas the rapid disruption of square arrays and the translational suppression by microRNAs are beneficial mechanisms to protect the brain from further insults.

After the initial energy depletion, ATP levels in cortical tissue recovered to about 75% of the normal level 4 hours after transient ischemia (95). The recovery of ATP synthesis should allow the restoration of ionic homeostasis. It has been shown that in transient ischemia, the depression of [Na^+^]_ex_ and increase in [K^+^]_ex_ are rapidly restored after the restoration of blood flow, suggesting that the clearance mechanism of ions is energy dependent (89). Since potassium siphoning through astrocytes is accompanied by water flux, AQP4 expression should benefit the fluid clearance phase, even though it exacerbates brain water entry during the formation of cytotoxic edema. This again highlights how a timely and short term inhibition may be immensely beneficial.

An elevation of [K^+^]_ex_ is reported to cause an influx of Na^+^ into the brain through Na^+^-K^+^ ATPase located in the capillary endothelium (96). In addition, the inflammatory response induces the disruption of the BBB, leading to the extravasation of plasma constituents into the brain, causing an elevation of brain osmolarity compared to the blood during vasogenic edema. The formation of vasogenic edema is illustrated in Figure 3C. However, it is not clear whether the hyperosmolarity is found within the ECS or the cytosol of the brain, which makes it challenging to study the microscopic route of brain water clearance during the progression and resolution of vasogenic edema. In the following section, we argue that the difference in *1) osmolarity gradients between the cytosol-ECS compartments* and *2) clearance routes of osmolytes in vasogenic and cytotoxic edema* are responsible for the opposing roles of AQP4 in these two types of edematous conditions.

### Vasogenic edema and the comparison of water transport in vasogenic vs cytotoxic edema

Vasogenic edema is caused by the extravasation of plasma constituents as a consequence of BBB breakdown. The disintegration of BBB is orchestrated by molecules including thrombin (97), VEGF (98-102), matrix metallopeptidase-9 (MMP-9) (98,103), and leukotrienes (81), many of which have an effect on AQP4 expression.

AQP4 up-regulation is observed during the disruption of BBB. The increased expression of VEGF is associated with the induction of hypoxia inducible factor-1α (HIF-1α) during hypoxic conditions, and HIF-1α is an inducer of AQP4 up-regulation through increased VEGF (104). In a model of TBI, AQP4 up-regulation was attributed to the increase in MMP-9 and HIF-α (22). AQP4 expression in the substantia nigra is increased by 10-fold following disruption of BBB in ovariectomized animals (105). The timing of BBB opening after ischemic injury has been measured based on the extravasation of a tracer (106,107). A significant increase in BBB permeability was found at 6 hours (91), 12 hours (108), or 48 hours (109) after permanent MCA occlusion. In many injury models of ischemia, the increase in AQP4 protein levels corresponds to the timing of BBB opening.

AQP4 facilitates water clearance in vasogenic edema (18), in contrast to its detrimental role in cytotoxic edema. We suggest that the seemingly opposite effects of AQP4 on the severity of cytotoxic and vasogenic edema may be caused by the difference in their osmotic environment and the direction of osmotic gradient-induced water flow. Since AQP4 channels passively conduct water molecules toward the compartment with the higher osmolarity (110), AQP4-mediated water flux in cytotoxic and vasogenic edema depends on the existence of osmolarity gradients. Above, we summarized evidence confirming the development of a hyperosmolar environment in the cytosol during cytotoxic edema, and the detrimental effects of AQP4.

Experimental evidence has already established that in vasogenic edema the brain compartment is hyperosmolar compared to the blood. However, there is no definitive evidence to confirm whether the increased osmolarity is due to accumulation of osmolytes in the ECS, or whether the hyperosmolar brain in vasogenic edema resembles the osmotic environment in cytotoxic edema, where the hyperosmolar compartment is the cytosol. Determining the relative osmolar relationship between the cytosol-ECS-blood compartments is important in establishing the direction and route of water clearance, and in understanding why the expression of AQP4 in vasogenic edema is beneficial.

There is a lack of a direct measurement of the respective osmolarities of the cytosol vs the ECS during vasogenic edema. Even though the following studies reported measurements in bulk brain tissue, they are useful in constructing a picture of the osmotic environment. In permanent ischemia, brain water content shows a high correlation with total sodium content (111) during vasogenic edema. The correlation between brain water content and total sodium content was confirmed in another report (108). In a permanent MCA occlusion in rats, brain water content was found to correlate with the increase in sodium and the decrease in potassium content in the total tissue, not the extravasation of albumin (109). Another study using permanent MCA occlusion confirmed that there was a net increase in cation content in the brain, which parallels the time course of elevated brain water content (91). The source of increased brain sodium content is likely due to the transport of sodium from blood to brain (112). While protein extravasation used to be considered the osmotic force driving vasogenic edema (113), the elevation of cations in the brain has been confirmed to play a dominant role (91). However, whether or not the increased sodium is found in the ECS or cytosol is not clear, since these studies measure the ion content of the processed bulk brain tissue without distinguishing the ECS and cytosol compartments. In addition, the edematous condition after permanent ischemia is both cytotoxic and vasogenic in nature, and the persistence of the cytotoxic component even after the opening of the BBB may explain the observed elevation of sodium. Therefore, the observed elevation of Na^+^ in these reports of permanent ischemia may be both extracellular and intracellular. Interestingly, a study using a cold-induced injury model of vasogenic edema reports measurements in the edema fluid of cats (114). They found an elevated colloidal osmotic pressure in the edema fluid; however, the sodium content did not experience any significant change in the edema fluid, which seems to suggest that the elevated colloidal osmotic pressure is due to other osmolytes. In addition to ionic accumulation in the brain, the extravasation of serum proteins into the ECS contributes to the ECS osmolarity. After cold-induced vasogenic edema, intravenously injected horseradish peroxidase leaks out of the vasculature into the ECS. Even though some HRP were taken up by cells including astrocytes, the intracellular HRP was seen in vacuoles or vesicles (115), which indicates that the HRP does not contribute to the osmolarity of the cytosol. Lastly, the ECS is enlarged in vasogenic edema, but it is greatly reduced in cytotoxic edema (116). The enlarged ECS in vasogenic edema is indicative of a higher osmolarity in the ECS compared to the cytosol, due to the passive nature of water transport. However, the effect of hydrostatic pressure from the fluid extravasation on the enlargement of the ECS cannot be ruled out. Since to the best of authors’ knowledge simultaneous measurements of ECS and intracellular osmolarity in vasogenic edema do not exist, the elevated osmolarity of the ECS in vasogenic edema remains a conjecture. However, regardless of whether the ECS is hyperosmolar to both the cytosol and the blood or both the ECS and cytosol are hyperosmolar to the blood, it is certain that the osmotic environment in vasogenic edema is very distinct from that found in cytotoxic edema, and this is key to understanding the opposing roles of AQP4 in these two forms of edema.

Based on this view, it is reasonable to propose that during vasogenic edema, astrocytes aid in osmolyte clearance from the ECS by enhanced water transfer through AQP4 channels. There may be multiple mechanisms of osmolyte clearance. Osmolytes can be cleared through the transvascular route into the blood (115). They may also be cleared though the interstitial fluid route into the ventricular system or transferred through the paravascular pathways into the cerebrospinal fluid pools (117-119). The significance of AQP4 to the clearance of molecules from the parenchyma was recently demonstrated in animals lacking AQP4 channels who showed a 70% reduction in protein clearance compared to normal (8). Therefore, to understand the routes of water clearance, it is indispensable to look at the difference in the pathways of osmolyte clearance in vasogenic and cytotoxic edema.

The second consideration regarding the difference in water clearance in vasogenic and cytotoxic edema is the type of osmolytes accumulated in the brain. A passive water flux accompanies the clearance of osmoles such as K^+^ (120). During vasogenic edema, it is proposed that the clearance of extravasated proteins occurs through both the transvascular route (115) and through CSF via the Virchow-Robin space (121). The recovery of the dysregulated osmolar environment can occur through the transport of ions by astrocytes from the injury core back into the bloodstream at a site with intact BBB away from the injury core. Astrocytes siphon K^+^ through this transcellular route, and the transport of water accompanies the siphoning of K^+^ (120), supporting the existence of a transcellular pathway of water clearance. The transcellular clearance is represented in Figure 3D. Evidence shows that water transport is mandatory for the efficiency of ion siphoning by brain astrocytes – the mislocalization of the perivascular AQP4 pool by α-syntrophin deletion delays K^+^ clearance, confirming the coupling of K^+^ siphoning and water transport (120). The tight coupling of AQP4-mediated water transport with ionic transport is critical in neuronal signal transduction as well as sensory signal transduction (122). More than likely, it is the pool of AQP4 in astroglial endfeet facing an intact BBB away from the injury core that is exerting this beneficial effects in brain water clearance. Experiments showed that while AQP4 at the perivascular pool at the injury core was decreased after ischemia, the cortical border zone showed a slight increase in AQP4 at the same time (47). In many other animal models of experimental ischemia, AQP4 up-regulation is seen in the border zone, supporting the idea that clearance of both osmoles and water may occur at a site away from the injury core. The proposed direction of water transport in cytotoxic in comparison to vasogenic edema is shown in Figure 3. In cytotoxic edema, the main disturbance in osmolar dysregulation is ionic imbalance between cytosol and ECS. In vasogenic edema, the osmolar dysregulation is caused by multiple forms of osmolytes including protein extravasation in addition to ions, requiring various routes of clearance. Therefore, whether or not AQP4 plays a beneficial role in a particular stage of injury depends on the directions of the osmolar gradients between the cell, extracellular space, and blood at a specific point in time and space (near vs far from the injury site), and the molecular route through which osmoles are cleared from the brain. To design effective therapies for water clearance, these two factors merit utmost consideration, and the clarification of the osmolar gradients and the solute clearance pathways are of high priority. This further highlights the importance of achieving optimal timing for pharmacological intervention targeting AQP4 channels, since the success of the therapy depends on the osmolar environment in the brain post-injury.

### Effect of osmotic stress on Aqp4 gene expression

AQP4 expression is up-regulated by osmotic stress. In terms of cellular signaling, osmotic stress activates *Aqp4* gene transcription in astrocyte cultures as well as in vivo (123). Hyperosmotic stress activates RAC1 (124,125) and up-regulates AQP4 through the p38 MAPK pathway (62,123) as shown in Figure 2. p38 translocates to the nucleus and phosphorylates c-JUN and c-FOS, forming AP-1 (126,127). Slight osmolarity elevation (324 mOsm) does not cause its transcriptional activation, while an osmolarity of 363 ± 2.7 mOsm stimulates a 1.5 fold increase in both *Aqp4* mRNA and protein (123), suggesting that there is a threshold for osmolarity-induced transcriptional activation. Based on electrode measurements, the transmembrane osmotic gradient resulting from the accumulation of Na^+^ and K^+^ ions alone could amount to 65 mEq/kg (87), without considering non-ionic osmolytes, which is sufficient to induce AQP4 transcriptional activation in the brain. Since transcriptional regulation of AQP4 as opposed to membrane translocation would lead to a change in AQP4 levels on a longer time-scale, it is likely that the induction of AQP4 transcription by the presence of osmotic stress in the brain is a physiologically protective response that aims to up-regulate AQP4 water channels during the re-establishment of ionic homeostasis.

### Effect of brain inflammatory cytokines and signaling molecules on Aqp4 gene expression

The breakdown of BBB promotes the spreading of inflammation. During inflammation, leukocytes are recruited to injury site and the increased permeability of BBB facilitates their extravasation. The secretion of both pro-inflammatory and anti-inflammatory cytokines by immune cells as well as neurons, astrocytes, and microglia modulates the spread and resolution of the inflammatory response. Inflammatory cytokines have both therapeutic and deleterious effects in the brain. It has been shown for astrocytes that their expression levels of AQP4 are highly inducible by cytokine exposure. As we have discussed previously, pro-inflammatory leukotrienes not only promote macromolecule extravasation at the BBB, but also up-regulate AQP4. Therefore, it is highly probable that an elevation of brain water permeability is a process that accompanies neuroinflammation.

Elevated levels of IL-10, TNF-α, IL-1β, and IL-2 are found after middle cerebral artery occlusion in the rat brain (128). An increase in TNF-α, IL-6, and IL-1β is found after transient cerebral ischemia (129). The surge of inflammatory cytokines in the brain can be detected within an hour after injury, as depicted in Figure 2. In astrocytes, IL-1β is a potent activator of NFκB (130), which leads to increased *AQP4* transcription. When inactive, NFκB is located in the cytoplasm, sequestered by its inhibitory molecule, IκB. IL-1β causes the increased degradation of IκB (67) and nuclear translocation of NFκB (131), where it induces the transcription of target genes. A putative binding site for NF-κB has been found near exon 1 of *Aqp4* gene (67), which regulates the induction of the transcription for the short M23 AQP4 isoform. Ito et al (67) confirmed that IL-1β was a potent inducer of AQP4 expression in astrocytes and that this induction was mediated by NF-κB activation. The activation of NFκB in astrocytes is mediated by PI3K and AKT signaling pathways after IL-1β stimulation (132). The mechanism of IL-1β induced *Aqp4* activation is shown in Figure 2. In addition to activating NFκB, IL-1β is shown to transcriptionally up-regulate C/EBPβ and C/EBPδ in astrocytes (133), which are immediate early genes for the acute phase response (134). The administration of IL-1β changes brain water permeability in vivo. The injection of IL-1β has been shown to exacerbate brain edema after ischemic injury in rats, even though IL-1β injection into healthy animals does not change brain water content (67,135). Injection of anti-IL-1β attenuated the ischemia-induced edema (135). The up-regulation of AQP4 by IL-1β in astrocytes could be partially responsible for edema worsening. We believe that the IL-1 induced up-regulation of AQP4 in a normal brain does not have deleterious effects on brain water regulation due to the lack of abnormal osmotic gradients in a healthy brain. Another cytokine, TNF-α, is also a potent activator of NFκB (136). Interestingly, TNF-α seems to interact with IL-1 in a positive-feedback system to induce the spread of inflammation (137,138). This positive feedback loop can cause a sustained elevation of cytokines. The up-regulation of AQP4 by TNF-α has been confirmed in an epithelial cell line (139). In addition, VEGF has been found to induce up-regulation of *Aqp4* mRNA in vivo (140). VEGF promotes angiogenesis and increases vascular permeability in the brain, and its transcript starts to increase as early as 2 hours after ischemic injury (141). Like the opposing effects of AQP4 in different edematous phases post-injury, VEGF injection has a detrimental role if injected 1 hour after injury, but promotes recovery when injected 48 hours after injury (140). We conjecture that the sustained rise of AQP4 levels observed in brain pathologies (Figure 1) is mainly caused by the elevation of cytokines during neuroinflammation.

### Transcriptional inhibition of AQP4 by thrombin via PKC signaling

Thrombin, a coagulation factor, is released from the blood stream during injury, but it can also be produced by brain cells (142) during ischemia (143) or spinal cord injury (144). Thrombin is also detected in the CSF after hemorrhage (145). It is a direct cause of edema formation in the brain (146). Contrary to its deleterious effects in the ischemic brain, the injection of thrombin prior to the injury could attenuate edema through a mechanism called thrombin preconditioning (147). In astrocytes, thrombin inhibits the transcription of *Aqp4* through the activation of PKC signaling pathway (148). The down-regulation of AQP4 by thrombin is partially mediated by the G protein coupled PAR-1 receptor, which is also known to activate ERK and JNK pathways in astrocytes (149). PKC activation down-regulates AQP4 in a time-dependent manner and PKC depletion caused by prolonged exposure reverses AQP4 down-regulation (148). This mechanism is portrayed in Figure 2. The activation of PKC by agents including TPA (150,151) and propofol (152) also down-regulates *Aqp4* mRNA (150). Since the down-regulation of AQP4 during cytotoxic edema is beneficial, and an early injection of thrombin could cause AQP4 down-regulation, it is possible that the protective effect of thrombin preconditioning is partially mediated through AQP4 down-regulation.

## Conclusions

1) Astrocytes control their membrane water permeability by different levels of regulatory mechanisms combining transcriptional, translational, and post-translational modification processes. Astrocytes dynamically regulate AQP4 expression levels, its subcellular membrane localization, isoform abundance, as well as square array integrity after neurological injury.

2) After neurological injury, astroglial membrane water permeability is probably regulated by a combination of these mechanisms in order to achieve both short and long term temporal effect, in addition to domain-specific regulation of AQP4 expression (endfeet membrane opposing BBB vs opposing ventricle). It will be interesting to test whether astrocytes modulate water regulation during injury and recovery periods by simultaneously utilizing these different levels of control.

3) A hypothesis was proposed that the opposing roles of AQP4 in cytotoxic and vasogenic edema could be explained by the difference in osmolar environments in the brain between the intracellular, extracellular, and blood compartments. During cytotoxic edema, the cytosol accumulates osmolytes, leading to a passive water influx. AQP4 is detrimental during cytotoxic edema. However, we propose that the osmotic environment during vasogenic edema is very different from that of cytotoxic edema, which is likely characterized by the elevated osmolarity in the extracellular space. During vasogenic edema, astrocytes play an important role in osmolyte and water clearance through the transcellular route, among other clearance mechanisms. The difference in osmolar gradients and water transport between vasogenic and cytotoxic edema underlies the opposing roles of AQP4 in these two forms of edema.

4) For therapy development aimed at improving brain water clearance during the edematous phase after neurological injury, the dosing times of the pharmacological agent need to be precisely planned. A short-term reduction in astrocyte water permeability for reducing cytotoxic swelling (by limiting brain water entry) and a long-term increase in AQP4 expression for facilitating the clearance of vasogenic edema are likely beneficial. Molecular therapies involving two or more therapeutic agents with different speed of action to satisfy the specific temporal profiles of brain water entry and water clearance can likely provide the maximal benefit in reducing edema.

## References

[1] Nielsen S, Nagelhus EA, Amiry-Moghaddam M, Bourque C, Agre P, Ottersen OP (1997). Specialized membrane domains for water transport in glial cells: High-resolution immunogold cytochemistry of aquaporin-4 in rat brain.. J Neurosci.

[2] Bloch O, Manley GT (2007). The role of aquaporin-4 in cerebral water transport and edema.. Neurosurg Focus.

[3] Binder DK, Yao XM, Zador Z, Sick TJ, Verkman AS, Manley GT (2006). Increased seizure duration and slowed potassium kinetics in mice lacking aquaporin-4 water channels.. Glia.

[4] Binder DK, Nagelhus EA, Ottersen OP (2012). Aquaporin-4 and epilepsy.. Glia.

[5] Benga G, Morariu VV (1977). Membrane Defect affecting water permeability in human epilepsy.. Nature.

[6] Nicaise C, Soyfoo MS, Authelet M, De Decker R, Bataveljic D, Delporte C (2009). Aquaporin-4 overexpression in rat ALS model.. Anat Rec.

[7] Rodriguez A, Perez-Gracia E, Espinosa JC, Pumarola M, Torres J, Ferrer I (2006). Increased expression of water channel aquaporin 1 and aquaporin 4 in Creutzfeldt-Jakob disease and in bovine spongiform encephalopathy-infected bovine-PrP transgenic mice.. Acta Neuropathol.

[8] Iliff JJ, Wang MH, Liao YH, Plogg BA, Peng WG, Gundersen GA (2012). A paravascular pathway facilitates CSF Flow through the brain parenchyma and the clearance of interstitial solutes, including amyloid beta.. Sci Transl Med.

[9] Solenov E, Watanabe H, Manley GT, Verkman AS (2004). Sevenfold-reduced osmotic water permeability in primary astrocyte cultures from AQP-4-deficient mice, measured by a fluorescence quenching method.. Am J Physiol Cell Physiol.

[10] Gunnarson E, Axehult G, Baturina G, Zelenin S, Zelenina M, Aperia A (2005). Lead induces increased water permeability in astrocytes expressing aquaporin 4.. Neuroscience.

[11] Nicchia GP, Frigeri A, Liuzzi GM, Svelto M (2003). Inhibition of aquaporin-4 expression in astrocytes by RNAi determines alteration in cell morphology, growth, and water transport and induces changes in ischemia-related genes.. FASEB J.

[12] Badaut J, Ashwal S, Adami A, Tone B, Recker R, Spagnoli D (2011). Brain water mobility decreases after astrocytic aquaporin-4 inhibition using RNA interference.. J Cereb Blood Flow Metab.

[13] Wang HW, Wang XM, Guo QY (2012). The correlation between DTI parameters and levels of AQP-4 in the early phases of cerebral edema after hypoxic-ischemic/reperfusion injury in piglets.. Pediatr Radiol.

[14] Zeynalov E, Chen CH, Froehner SC, Adams ME, Ottersen OP, Amiry-Moghaddam M (2008). The perivascular pool of aquaporin-4 mediates the effect of osmotherapy in postischemic cerebral edema.. Crit Care Med.

[15] Manley GT, Fujimura M, Ma T, Noshita N, Filiz F, Bollen AW (2000). Aquaporin-4 deletion in mice reduces brain edema after acute water intoxication and ischemic stroke.. Nat Med.

[16] Yang BX, Zador Z, Verkman AS (2008). Glial cell aquaporin-4 overexpression in transgenic mice accelerates cytotoxic brain swelling.. J Biol Chem.

[17] Haj-Yasein NN, Vindedal GF, Eilert-Olsen M, Gundersen GA, Skare O, Laake P (2011). Glial-conditional deletion of aquaporin-4 (Aqp4) reduces blood-brain water uptake and confers barrier function on perivascular astrocyte endfeet.. Proc Natl Acad Sci U S A.

[18] Papadopoulos MC, Manley GT, Krishna S, Verkman AS (2004). Aquaporin-4 facilitates reabsorption of excess fluid in vasogenic brain edema.. FASEB J.

[19] Agre P, Nielsen S, Ottersen OP (2004). Towards a molecular understanding of water homeostasis in the brain.. Neuroscience.

[20] Migliati ER, Amiry-Moghaddam M, Froehner SC, Adams ME, Ottersen OP, Bhardwaj A (2010). Na+-K+-2Cl(-) cotransport inhibitor attenuates cerebral edema following experimental stroke via the perivascular pool of aquaporin-4.. Neurocrit Care.

[21] Furman CS, Gorelick-Feldman DA, Davidson KGV, Yasumura T, Neely JD, Agre P (2003). Aquaporin-4 square array assembly: Opposing actions of M1 and M23 isoforms.. Proc Natl Acad Sci U S A.

[22] Higashida T, Kreipke CW, Rafols JA, Peng CY, Schafer S, Schafer P (2011). The role of hypoxia-inducible factor-la, aquaporin-4, and matrix metalloproteinase-9 in blood-brain barrier disruption and brain edema after traumatic brain injury. Laboratory investigation.. J Neurosurg.

[23] Ren Z, Iliff JJ, Yang LJ, Yang JK, Chen XL, Chen MJ (2013). 'Hit & Run' model of closed-skull traumatic brain injury (TBI) reveals complex patterns of post-traumatic AQP4 dysregulation.. J Cereb Blood Flow Metab.

[24] Sun MC, Honey CR, Berk C, Wong NLM, Tsui JKC (2003). Regulation of aquaporin-4 in a traumatic brain injury model in rats.. J Neurosurg.

[25] Madathil SK, Nelson PT, Saatman KE, Wilfred BR (2011). MicroRNAs in CNS injury: Potential roles and therapeutic implications.. BioEssays.

[26] Ke C, Poon WS, Ng HK, Pang JCS, Chan Y (2001). Heterogeneous responses of aquaporin-4 in oedema formation in a replicated severe traumatic brain injury model in rats.. Neurosci Lett.

[27] Guo Q, Sayeed I, Baronne LM, Hoffman SW, Guennoun R, Stein DG (2006). Progesterone administration modulates AQP4 expression and edema after traumatic brain injury in male rats.. Exp Neurol.

[28] Fukuda AM, Pop V, Spagnoli D, Ashwal S, Obenaus A, Badaut J (2012). Delayed increase of astrocytic aquaporin 4 after juvenile traumatic brain injury: possible role in edema resolution?. Neuroscience.

[29] Kiening KL, van Landeghem FKH, Schreiber S, Thomale UW, von Deimling A, Unterberg AW (2002). Decreased hemispheric Aquaporin-4 is linked to evolving brain edema following controlled cortical impact injury in rats.. Neurosci Lett.

[30] Stroop R, Thomale UW, Pauser S, Bernarding J, Vollmann W, Wolf KJ (1998). Magnetic resonance imaging studies with cluster algorithm for characterization of brain edema after controlled cortical impact injury (CCII).. Acta Neurochir Suppl.

[31] Barzo P, Marmarou A, Fatouros P, Hayasaki K, Corwin F (1997). Contribution of vasogenic and cellular edema to traumatic brain swelling measured by diffusion-weighted imaging.. J Neurosurg.

[32] Zhao J, Moore AN, Clifton GL, Dash PK (2005). Sulforaphane enhances aquaporin-4 expression and decreases cerebral edema following traumatic brain injury.. J Neurosci Res.

[33] Taupin V, Toulmond S, Serrano A, Benavides J, Zavala F (1993). Increase in IL-6, IL-1 and TNF levels in rat-brain following traumatic lesion - Influence of pre-traumatic and posttraumatic treatment with Ro5 4864, a peripheral-type (P-Site) benzodiazepine ligand.. J Neuroimmunol.

[34] Stover JF, Schoning B, Beyer TF, Woiciechowsky C, Unterberg AW (2000). Temporal profile of cerebrospinal fluid glutamate, interleukin-6, and tumor necrosis factor-alpha in relation to brain edema and contusion following controlled cortical impact injury in rats.. Neurosci Lett.

[35] McClain CJ, Cohen D, Ott L, Dinarello CA, Young B (1987). Ventricular fluid interleukin-1 activity in patients with head injury.. J Lab Clin Med.

[36] Del Bigio MR, Enno TL (2008). Effect of hydrocephalus on rat brain extracellular compartment.. Cerebrospinal Fluid Res.

[37] Skjolding AD, Rowland IJ, Sogaard LV, Praetorius J, Penkowa M, Juhler M (2010). Hydrocephalus induces dynamic spatiotemporal regulation of aquaporin-4 expression in the rat brain.. Cerebrospinal Fluid Res.

[38] Tourdias T, Dragonu I, Fushimi Y, Deloire MSA, Boiziau C, Brochet B (2009). Aquaporin 4 correlates with apparent diffusion coefficient and hydrocephalus severity in the rat brain: A combined MRI-histological study.. Neuroimage.

[39] Feng X, Papadopoulos MC, Liu J, Li L, Zhang D, Zhang H (2009). Sporadic obstructive hydrocephalus in Aqp4 null mice.. J Neurosci Res.

[40] Bloch O, Auguste KI, Manley GT, Verkman AS (2006). Accelerated progression of kaolin-induced hydrocephalus in aquaporin-4-deficient mice.. J Cereb Blood Flow Metab.

[41] Tarkowski E, Tullberg M, Fredman P, Wikkelso C (2003). Normal pressure hydrocephalus triggers intrathecal production of TNF-alpha.. Neurobiol Aging.

[42] Cacabelos R, Barquero M, Garcia P, Alvarez XA, Varela de Seijas E (1991). Cerebrospinal fluid interleukin-1 beta (IL-1 beta) in Alzheimer's disease and neurological disorders.. Methods Find Exp Clin Pharmacol.

[43] Ribeiro MC, Hirt L, Bogousslavsky J, Regli L, Badaut J (2006). Time course of aquaporin expression after transient focal cerebral ischemia in mice.. J Neurosci Res.

[44] Lo ACY, Chen AYS, Hung VKL, Yaw LP, Fung MKL, Ho MCY (2005). Endothelin-1 overexpression leads to further water accumulation and brain edema after middle cerebral artery occlusion via aquaporin 4 expression in astrocytic end-feet.. J Cereb Blood Flow Metab.

[45] Badaut J, Ashwal S, Tone B, Regli L, Tian HR, Obenaus A (2007). Temporal and regional evolution of aquaporin-4 expression and magnetic resonance imaging in a rat pup model of neonatal stroke.. Pediatr Res.

[46] Hirt L, Ternon B, Price M, Mastour N, Brunet JF, Badaut J (2009). Protective role of early aquaporin 4 induction against postischemic edema formation.. J Cereb Blood Flow Metab.

[47] Frydenlund DS, Bhardwaj A, Otsuka T, Mylonakou MN, Yasumura T, Davidson KG (2006). Temporary loss of perivascular aquaporin-4 in neocortex after transient middle cerebral artery occlusion in mice.. Proc Natl Acad Sci U S A.

[48] Cuevas P, Gutierrez Diaz JA, Dujovny M, Diaz FG, Ausman JI (1985). Disturbance of plasmalemmal astrocytic assemblies in focal and selective cerebral ischemia.. Anat Embryol (Berl).

[49] Suzuki M, Iwasaki Y, Yamamoto T, Konno H, Yoshimoto T, Suzuki J (1984). Disintegration of orthogonal arrays in perivascular astrocytic processes as an early event in acute global-ischemia.. Brain Res.

[50] Amiry-Moghaddam M, Xue R, Haug F-M, Neely JD, Bhardwaj A, Agre P (2004). Alpha-syntrophin deletion removes the perivascular but not endothelial pool of aquaporin-4 at the blood–brain barrier and delays the development of brain edema in an experimental model of acute hyponatremia.. FASEB J.

[51] Finkel T (2003). Oxidant signals and oxidative stress.. Curr Opin Cell Biol.

[52] Simon HU, Haj-Yehia A, Levi-Schaffer F (2000). Role of reactive oxygen species (ROS) in apoptosis induction.. Apoptosis.

[53] Samali A, Nordgren H, Zhivotovsky B, Peterson E, Orrenius S (1999). A comparative study of apoptosis and necrosis in HepG2 cells: Oxidant-induced caspase inactivation leads to necrosis.. Biochem Biophys Res Commun.

[54] Tyurin VA, Tyurina YY, Borisenko GG, Sokolova TV, Ritov VB, Quinn PJ (2000). Oxidative stress following traumatic brain injury in rats: Quantitation of biomarkers and detection of free radical intermediates.. J Neurochem.

[55] Hall ED, Detloff MR, Johnson K, Kupina NC (2004). Peroxynitrite-mediated protein nitration and lipid peroxidation in a mouse model of traumatic brain injury.. J Neurotrauma.

[56] Cernak I, Savic VJ, Kotur J, Prokic V, Veljovic M, Grbovic D (2000). Characterization of plasma magnesium concentration and oxidative stress following graded traumatic brain injury in humans.. J Neurotrauma.

[57] Lewen A, Hillered L (1998). Involvement of reactive oxygen species in membrane phospholipid breakdown and energy perturbation after traumatic brain injury in the rat.. J Neurotrauma.

[58] Azbill RD, Mu XJ (1997). BruceKeller AJ, Mattson MP, Springer JE. Impaired mitochondrial function, oxidative stress and altered antioxidant enzyme activities following traumatic spinal cord injury.. Brain Res.

[59] Childs EW, Udobi KF, Wood JG, Hunter FA, Smalley DM, Cheung LY (2002). In vivo visualization of reactive oxidants and leukocyte-endothelial adherence following hemorrhagic shock.. Shock.

[60] Rao GN, Glasgow WC, Eling TE, Runge MS (1996). Role of hydroperoxyeicosatetraenoic acids in oxidative stress-induced activating protein 1 (AP-1) activity.. J Biol Chem.

[61] Maki A, Berezesky IK, Fargnoli J, Holbrook NJ, Trump BF (1992). Role of [Ca2+]i in induction of c-fos, c-jun, and c-myc mRNA in rat PTE after oxidative stress.. FASEB J.

[62] Pan CF, Zhu SM, Zheng YY (2010). Ammonia induces upregulation of aquaporin-4 in neocortical astrocytes of rats through the p38 mitogen-activated protein kinase pathway.. Chinese Med J (Engl)..

[63] Rosenberger J, Petrovics G, Buzas B (2001). Oxidative stress induces proorphanin FQ and proenkephalin gene expression in astrocytes through p38- and ERK-MAP kinases and NF-kappaB.. J Neurochem.

[64] Schreck R, Albermann K, Baeuerle PA (1992). Nuclear factor kappa-B - an oxidative stress-responsive transcription factor of eukaryotic cells (a review).. Free Radic Res Commun.

[65] Vile GF, Tanewiliitschew A, Tyrrell RM (1995). Activation of NF-kappa-B in human skin fibroblasts by the oxidative stress generated by uva radiation.. Photochem Photobiol.

[66] Adcock IM, Brown CR, Kwon O, Barnes PJ (1994). Oxidative stress induces NF kappa B DNA binding and inducible NOS mRNA in human epithelial cells.. Biochem Biophys Res Commun.

[67] Ito H, Yamamoto N, Arima H, Hirate H, Morishima T, Umenishi F (2006). Interleukin-1 beta induces the expression of aquaporin-4 through a nuclear factor-kappa B pathway in rat astrocytes.. J Neurochem.

[68] Kang KW, Lee SJ, Kim SG (2005). Molecular mechanism of Nrf2 activation by oxidative stress.. Antioxid Redox Signal.

[69] Kang KW, Lee SJ, Park JW, Kim SG (2002). Phosphatidylinositol 3-kinase regulates nuclear translocation of NF-E2-related factor 2 through actin rearrangement in response to oxidative stress.. Mol Pharmacol.

[70] Mao L, Wang HD, Pan H, Qiao L (2011). Sulphoraphane enhances aquaporin-4 expression and decreases spinal cord oedema following spinal cord injury.. Brain Inj.

[71] Balboa MA, Balsinde J (2006). Oxidative stress and arachidonic acid mobilization. Biochim Biophys Acta.

[72] Baena RRY, Gaetani P, Paoletti P (1988). A study on cisternal CSF levels of arachidonic-acid metabolites after aneurysmal subarachnoid hemorrhage.. J Neurol Sci.

[73] Moskowitz MA, Kiwak KJ, Hekimian K, Levine L (1984). Synthesis of compounds with properties of leukotrienes C4 and D4 in gerbil brains after ischemia and reperfusion.. Science.

[74] Gaetani P, Marzatico F, Rodriguez y Baena R, Pacchiarini L, Vigano T, Grignani G (1990). Arachidonic acid metabolism and pathophysiologic aspects of subarachnoid hemorrhage in rats.. Stroke.

[75] Farias S, Frey LC, Murphy RC, Heidenreich KA (2009). Injury-Related production of cysteinyl leukotrienes contributes to brain damage following experimental traumatic brain injury.. J Neurotrauma.

[76] Schuhmann MU, Mokhtarzadeh M, Stichtenoth DO, Skardelly M, Klinge PA, Gutzki FM (2003). Temporal profiles of cerebrospinal fluid leukotrienes, brain edema and inflammatory response following experimental brain injury.. Neurol Res.

[77] Dhillon HS, Dose JM, Prasad MR (1996). Regional generation of leukotriene C4 after experimental brain injury in anesthetized rats.. J Neurotrauma.

[78] Kiwak KJ, Moskowitz MA, Levine L (1985). Leukotriene Production in Gerbil Brain after Ischemic Insult, Subarachnoid Hemorrhage, and Concussive Injury.. J Neurosurg.

[79] Paoletti P, Gaetani P, Grignani G, Pacchiarini L, Silvani V, Rodriguez y Baena R (1988). CSF leukotriene C4 following subarachnoid hemorrhage.. J Neurosurg.

[80] Shimizu T, Watanabe T, Asano T, Seyama Y, Takakura K (1988). Activation of the arachidonate 5-lipoxygenase pathway in the canine basilar artery after experimental subarachnoidal hemorrhage.. J Neurochem.

[81] Lehr HA, Guhlmann A, Nolte D, Keppler D, Messmer K (1991). Leukotrienes as mediators in ischemia-reperfusion injury in a microcirculation model in the hamster.. J Clin Invest.

[82] Qi LL, Fang SH, Shi WZ, Huang XQ, Zhang XY, Lu YB (2011). CysLT(2) receptor-mediated AQP4 up-regulation is involved in ischemic-like injury through activation of ERK and p38 MAPK in rat astrocytes.. Life Sci.

[83] Bhattacharya P, Pandey AK, Paul S, Patnaik R, Yavagal DR (2013). Aquaporin-4 inhibition mediates piroxicam-induced neuroprotection against focal cerebral ischemia/reperfusion injury in rodents.. PLoS ONE.

[84] Roof RL, Hall ED (2000). Gender differences in acute CNS trauma and stroke: Neuroprotective effects of estrogen and progesterone.. J Neurotrauma.

[85] Xiao G, Wei J, Yan WQ, Wang WM, Lu ZH (2008). Improved outcomes from the administration of progesterone for patients with acute severe traumatic brain injury: a randomized controlled trial.. Crit Care.

[86] Habib P, Dang J, Slowik A, Victor M, Beyer C (2014). Hypoxia-Induced gene expression of aquaporin-4, cyclooxygenase-2 and hypoxia-inducible factor 1 alpha in rat cortical astroglia is inhibited by 17 beta-estradiol and progesterone.. Neuroendocrinology.

[87] Hossmann KA, Sakaki S, Zimmerman V (1977). Cation activities in reversible ischemia of the cat brain.. Stroke.

[88] Katayama Y, Kawamata T, Tamura T, Hovda DA, Becker DP, Tsubokawa T (1991). Calcium-dependent glutamate release concomitant with massive potassium flux during cerebral-ischemia in vivo.. Brain Res.

[89] Stiefel MF, Tomita Y, Marmarou A (2005). Secondary ischemia impairing the restoration of ion homeostasis following traumatic brain injury.. J Neurosurg.

[90] Yang GY, Chen SF, Kinouchi H, Chan PH, Weinstein PR (1992). Edema, cation content, and atpase activity after middle cerebral-artery occlusion in rats.. Stroke.

[91] Menzies SA, Betz AL, Hoff JT (1993). Contributions of Ions and Albumin to the Formation and Resolution of Ischemic Brain Edema.. J Neurosurg.

[92] Gunnarson E, Zelenina M, Axehult G, Song Y, Bondar A, Krieger P (2008). Identification of a molecular target for glutamate regulation of astrocyte water permeability.. Glia.

[93] Assentoft M, Kaptan S, Fenton R, Hua SZ, de Groot B, MacAulay N (2013). Phosphorylation of aquaporin-4 at Ser111 is Not required for channel gating. Glia.

[94] Kimelberg HK (1995). Current concepts of brain edema - review of laboratory investigations.. J Neurosurg.

[95] Levy DE, Duffy TE (1977). Cerebral energy-metabolism during transient ischemia and recovery in gerbil.. J Neurochem.

[96] Schielke GP, Moises HC, Betz AL (1990). Potassium activation of the Na,K-pump in isolated brain microvessels and synaptosomes.. Brain Res.

[97] Lee KR, Kawai N, Kim S, Sagher O, Hoff JT (1997). Mechanisms of edema formation after intracerebral hemorrhage: Effects of thrombin on cerebral blood flow, blood-brain barrier permeability, and cell survival in a rat model.. J Neurosurg.

[98] Valable S, Montaner J, Bellail A, Berezowski V, Brillault J, Cecchelli R (2005). VEGF-induced BBB permeability is associated with an MMP-9 activity increase in cerebral ischemia: both effects decreased by Ang-1.. J Cereb Blood Flow Metab.

[99] Argaw AT, Gurfein B, Zhang Y, Zameer A, John G (2009). VEGF-mediated disruption of endothelial CLN-5 promotes blood-brain barrier breakdown. Proc Natl Acad Sci U S.

[100] Proescholdt MA, Heiss JD, Walbridge S, Muhlhauser J, Capogrossi MC, Oldfield EH (1999). Vascular endothelial growth factor (VEGF) modulates vascular permeability and inflammation in rat brain.. J Neuropathol Exp Neurol.

[101] Zhang ZG, Zhang L, Jiang Q, Zhang RL, Davies K, Powers C (2000). VEGF enhances angiogenesis and promotes blood-brain barrier leakage in the ischemic brain.. J Clin Invest.

[102] Mayhan WG (1999). VEGF increases permeability of the blood-brain barrier via a nitric oxide synthase/cGMP-dependent pathway.. Am J Physiol.

[103] Fujimura M, Gasche Y, Morita-Fujimura Y, Massengale J, Kawase M, Chan PH (1999). Early appearance of activated matrix metalloproteinase-9 and blood-brain barrier disruption in mice after focal cerebral ischemia and reperfusion.. Brain Res.

[104] Wang YF, Fan ZK, Cao Y, Yu DS, Zhang YQ, Wang YS (2011). 2-Methoxyestradiol inhibits the up-regulation of AQP4 and AQP1 expression after spinal cord injury.. Brain Res.

[105] Tomas-Camardiel M, Venero JL, Herrera AJ, De Pablos RM, Pintor-Toro JA, Machado A (2005). Blood-brain barrier disruption highly induces aquaporin-4 mRNA and protein in perivascular and parenchymal astrocytes: protective effect by estradiol treatment in ovariectomized animals.. J Neurosci Res.

[106] Martz D, Beer M, Betz AL (1990). Dimethylthiourea reduces ischemic brain edema without affecting cerebral blood-flow.. J Cereb Blood Flow Metab.

[107] Betz AL, Coester HC (1990). Effect of steroids on edema and sodium uptake of the brain during focal ischemia in rats.. Stroke.

[108] Hatashita S, Hoff JT (1990). Brain edema and cerebrovascular permeability during cerebral-ischemia in rats.. Stroke.

[109] Gotoh O, Asano T, Koide T, Takakura K (1985). Ischemic Brain edema following occlusion of the middle cerebral-artery in the rat. 1. the time courses of the brain water, sodium and potassium contents and blood-brain-barrier permeability to i-125 albumin.. Stroke.

[110] Agre P, Bonhivers M, Borgnia MJ (1998). The aquaporins, blueprints for cellular plumbing systems.. J Biol Chem.

[111] Ito U, Ohno K, Nakamura R, Suganuma F, Inaba Y (1979). Brain edema during ischemia and after restoration of blood flow. Measurement of water, sodium, potassium content and plasma protein permeability.. Stroke.

[112] Schielke GP, Moises HC, Betz AL (1991). Blood to brain sodium-transport and interstitial fluid potassium concentration during early focal ischemia in the rat.. J Cereb Blood Flow Metab.

[113] Go K, Houthoff H, Hartsuiker J, Van der Molen-Woldendorp D, Zuiderveen F, Teelken A. Exudation of plasma protein fractions in vasogenic brain edema. Brain edema. Berlin, Heidelberg, New York, Tokyo: Springer;1985.

[114] Gazendam J, Go KG, Vanzanten AK (1979). Composition of isolated edema fluid in cold-induced brain edema.. J Neurosurg.

[115] Vorbrodt AW, Lossinsky AS, Wisniewski HM, Suzuki R, Yamaguchi T, Masaoka H (1985). Ultrastructural observations on the transvascular route of protein removal in vasogenic brain edema.. Acta Neuropathol.

[116] Papadopoulos MC, Binder DK, Verkman AS (2005). Enhanced macromolecular diffusion in brain extracellular space in mouse models of vasogenic edema measured by cortical surface photobleaching.. FASEB J.

[117] Nakada T (2014). Virchow-Robin space and aquaporin-4: new insights on an old friend.. Croat Med J.

[118] Rennels ML, Blaumanis OR, Grady PA (1990). Rapid solute transport throughout the brain via paravascular fluid pathways.. Adv Neurol.

[119] Rangroo Thrane V, Thrane AS, Plog BA, Thiyagarajan M, Iliff JJ, Deane R (2013). Paravascular microcirculation facilitates rapid lipid transport and astrocyte signaling in the brain.. Sci Rep.

[120] Amiry-Moghaddam M, Williamson A, Palomba M, Eid T, de Lanerolle NC, Nagelhus EA (2003). Delayed K+ clearance associated with aquaporin-4 mislocalization: Phenotypic defects in brains of alpha-syntrophin-null mice.. Proc Natl Acad Sci U S A.

[121] Reulen HJ, Tsuyumu M, Tack A, Fenske AR, Prioleau GR (1978). Clearance of edema fluid into cerebrospinal fluid: A mechanism for resolution of vasogenic brain edema.. J Neurosurg.

[122] Manley GT, Binder DK, Papadopoulos MC, Verkman AS (2004). New insights into water transport and edema in the central nervous system from phenotype analysis of aquaporin-4 null mice.. Neuroscience.

[123] Arima H, Yamamoto N, Sobue K, Umenishi F, Tada T, Katsuya H (2003). Hyperosmolar mannitol stimulates expression of aquaporins 4 and 9 through a p38 mitogen-activated protein kinase-dependent pathway in rat astrocytes.. J Biol Chem.

[124] Uhlik MT, Abell AN, Johnson NL, Sun W, Cuevas BD, Lobel-Rice KE (2003). Rac-MEKK3-MKK3 scaffolding for p38 MAPK activation during hyperosmotic shock.. Nat Cell Biol.

[125] Lewis A, Ciano CD, Rotstein OD, Kapus A (2002). Osmotic stress activates Rac and Cdc42 in neutrophils: role in hypertonicity-induced actin polymerization.. Am J Physiol Cell Physiol.

[126] Tanos T, Marinissen MJ, Leskow FC, Hochbaum D, Martinetto H, Gutkind JS (2005). Phosphorylation of c-fos by members of the p38 MAPK family - Role in the AP-1 response to UV light.. J Biol Chem.

[127] Humar M, Loop T, Schmidt R, Hoetzel A, Roesslein M, Andriopoulos N (2007). The mitogen-activated protein kinase p38 regulates activator protein 1 by direct phosphorylation of c-Jun.. Int J Biochem Cell Biol.

[128] Zhai QH, Futrell N, Chen FJ (1997). Gene expression of IL-10 in relationship to TNF-alpha, IL-1 beta and IL-2 in the rat brain following middle cerebral artery occlusion.. J Neurol Sci.

[129] Saito K, Suyama K, Nishida K, Sei Y, Basile AS (1996). Early increases in TNF-alpha, IL-6 and IL-1 beta levels following transient cerebral ischemia in gerbil brain.. Neurosci Lett.

[130] Moynagh PN, Williams DC, Oneill LAJ (1993). Interleukin-1 activates transcription factor NF-kappa-B in glial-cells.. Biochem J.

[131] Baldwin AS (1996). The NF-kappa B and I kappa B proteins: New discoveries and insights.. Annu Rev Immunol.

[132] Pousset F, Dantzer R, Kelley KW, Parnet P (2000). Interleukin-1 signaling in mouse astrocytes involves Akt: a study with interleukin-4 and IL-10.. Eur Cytokine Netw.

[133] Cardinaux JR, Allaman I, Magistretti PJ (2000). Pro-inflammatory cytokines induce the transcription factors C/EBP beta and C/EBP delta in astrocytes.. Glia.

[134] Hanson RW (1998). Biological role of the isoforms of C/EBP minireview series.. J Biol Chem.

[135] Yamasaki Y, Matsuura N, Shozuhara H, Onodera H, Itoyama Y, Kogure K (1995). Interleukin-1 as a pathogenetic mediator of ischemic brain-damage in rats.. Stroke.

[136] Han Y, He T, Huang DR, Pardo CA, Ransohoff RM (2001). TNF-alpha mediates SDF-1 alpha-induced NF-kappa B activation and cytotoxic effects in primary astrocytes.. J Clin Invest.

[137] Krueger JM, Obal F, Fang JD, Kubota T, Taishi P (2001). The role of cytokines in physiological sleep regulation.. Ann N Y Acad Sci.

[138] Granowitz EV, Vannier E, Poutsiaka DD, Dinarello CA (1992). Effect of interleukin-1 (Il-1) Blockade on cytokine synthesis. 2. Il-1 Receptor antagonist inhibits lipopolysaccharide-induced cytokine synthesis by human monocytes.. Blood.

[139] Janssens S, Caspers L, Willermain F, Delporte C. (2007). TNF-alpha and Interferon-gamma upregulate AQP4 protein expression in ARPE-19, a human retinal pigmented epithelial cell line. Acta Ophthalmologica.

[140] Rite I, Machado A, Cano J, Venero JL (2008). Intracerebral VEGF injection highly upregulates AQP4 mRNA and protein in the perivascular space and glia limitans extema.. Neurochem Int.

[141] Zhang ZG, Zhang L, Tsang W, Soltanian-Zadeh H, Morris T, Zhang R (2002). Correlation of VEGF and angiopoietin expression with disruption of blood-brain barrier and angiogenesis after focal cerebral ischemia.. J Cereb Blood Flow Metab.

[142] Dihanich M, Kaser M, Reinhard E, Cunningham D, Monard D (1991). Prothrombin mRNA is expressed by cells of the nervous system.. Neuron.

[143] Riek-Burchardt M, Striggow F, Henrich-Noack P, Reiser G, Reymann KG (2002). Increase of prothrombin-mRNA after global cerebral ischemia in rats, with constant expression of protease nexin-1 and protease-activated receptors.. Neurosci Lett.

[144] Citron BA, Smirnova IV, Arnold PM, Festoff BW (2000). Upregulation of neurotoxic serine proteases, prothrombin, and protease-activated receptor 1 early after spinal cord injury.. J Neurotrauma.

[145] Suzuki M, Kudo A, Otawara Y, Hirashima Y, Takaku A, Ogawa A (1999). Extrinsic pathway of blood coagulation and thrombin in the cerebrospinal fluid after subarachnoid hemorrhage.. Neurosurgery.

[146] Lee KR, Betz AL, Kim S, Keep RF, Hoff JT (1996). The role of the coagulation cascade in brain edema formation after intracerebral hemorrhage. Acta Neurochir (Wien)..

[147] Masada T, Xi GH, Hua Y, Keep RF (2000). The effects of thrombin preconditioning on focal cerebral ischemia in rats.. Brain Res.

[148] Tang Y, Cai DF, Chen YP (2007). Thrombin inhibits aquaporin 4 expression through protein kinase C-dependent pathway in cultured astrocytes.. J Mol Neurosci.

[149] Sorensen SD, Nicole O, Peavy RD, Montoya LM, Lee CJ, Murphy TJ (2003). Common signaling pathways link activation of murine PAR-1, LPA, and S1P receptors to proliferation of astrocytes.. Mol Pharmacol.

[150] Nakahama K, Nagano M, Fujioka A, Shinoda K, Sasaki H (1999). Effect of TPA on aquaporin 4 mRNA expression in cultured rat astrocytes.. Glia.

[151] Yamamoto N, Sobue K, Miyachi T, Inagaki M, Miura Y, Katsuya H (2001). Differential regulation of aquaporin expression in astrocytes by protein kinase C.. Brain Res Mol Brain Res.

[152] Zhu SM, Xiong XX, Zheng YY, Pan CF (2009). Propofol inhibits aquaporin 4 expression through a protein kinase C-dependent pathway in an astrocyte model of cerebral ischemia/reoxygenation.. Anesth Analg.

[153] Funahashi A, Morohashi M, Kitano H, Tanimura N (2003). CellDesigner: a process diagram editor for gene-regulatory and biochemical networks.. BIOSILICO.

